# NiO nanoparticle-decorated SnO_2_ nanosheets for ethanol sensing with enhanced moisture resistance

**DOI:** 10.1038/s41378-019-0060-7

**Published:** 2019-05-20

**Authors:** Gaoqiang Niu, Changhui Zhao, Huimin Gong, Zhitao Yang, Xiaohui Leng, Fei Wang

**Affiliations:** 1 School of Microelectronics, Southern University of Science and Technology, Shenzhen, 518055 China; 2 Department of Electrical and Electronic Engineering, Southern University of Science and Technology, Shenzhen, 518055 China

**Keywords:** Sensors, Electronic properties and materials

## Abstract

In a high relative humidity (RH) environment, it is challenging for ethanol sensors to maintain a high response and excellent selectivity. Herein, tetragonal rutile SnO_2_ nanosheets decorated with NiO nanoparticles were synthesized by a two-step hydrothermal process. The NiO-decorated SnO_2_ nanosheet-based sensors displayed a significantly improved sensitivity and excellent selectivity to ethanol gas. For example, the 3 mol% NiO-decorated SnO_2_ (SnO_2_-3Ni) sensor reached its highest response (153 at 100 ppm) at an operating temperature of 260 °C. Moreover, the SnO_2_-3Ni sensor had substantially improved moisture resistance. The excellent properties of the sensors can be attributed to the uniform dispersion of the NiO nanoparticles on the surface of the SnO_2_ nanosheets and the formation of NiO-SnO_2_ p–n heterojunctions. Considering the long-term stability and reproducibility of these sensors, our study suggests that the NiO nanoparticle-decorated SnO_2_ nanosheets are a promising material for highly efficient detection of ethanol.

## Nanomaterials: Novel material offers improved ethanol sensing

Researchers in China have developed a new material which is a more sensitive and selective ethanol sensor than existing technologies and which maintains its performance at high humidity. Fei Wang and colleagues at the Southern University of Science and Technology improved the sensing activity of tin-oxide nanosheets by decorating them with nickel-oxide nanoparticles. This altered the change in resistance in response to ethanol gas, increasing the material’s sensitivity to ethanol. The sensor was also selective, responding more strongly to ethanol than to methanol, acetone, or other gases. These decorated nanosheets maintained their performance over four weeks of testing and retained 71% of their response at humidity levels that reduced the performance of tin-oxide alone to 32%. These findings demonstrated that nickel-oxide decorated tin-oxide nanosheets are a promising material for improved ethanol sensors.

## Introduction

Metal oxide semiconductors (MOX) have attracted substantial attention in the field of gas detection over the past few decades due to their ease of use and reproducible response to various gases^1–3^. As a representative n-type MOX, SnO_2_ has been extensively investigated and used for commercial gas detectors^4^. To further improve the sensor performance, diverse SnO_2_-based nanostructures, such as nanoparticles^5^, nanosheets^6^, nanowires^7^, nanotubes^8^, hollow spheres^9^, and some hierarchical architectures^10–12^, have been developed. In these reports, two-dimensional (2D) SnO_2_ nanostructures exhibit a rather high catalytic activity on certain surface sites, which promotes their sensing performance^1^. On the other hand, SnO_2_-based sensors can also be substantially improved by the addition of appropriate dopants, such as Pd^13^, Sb^14^, Ce^15^, and Ni^16^. The gas sensing mechanisms related to doping effects, junction forming, surface catalytic effects, and synergistic effects have been explored to explain the improved sensor performance^17,18^. Among them, NiO is often used as a catalyst, which may also form p–n heterojunctions between the interface of the NiO and the SnO_2_^19,20^. In particular, a p-type NiO enables an increase in the oxygen adsorption that can react with target gases^21^.

According to previous studies, NiO-decorated SnO_2_ nanostructures were synthesized by various methods with beneficial ethanol sensing effects. NiO/SnO_2_ composite nanofibers prepared via electrospinning were used for ethanol detection, and a response up to 25.5 (100 ppm) was achieved at 300 °C, which was 12.7 times larger than that of the pure SnO_2_ nanofibers^19^. The ultrafine NiO/SnO_2_ nanoparticles obtained by thermal treatment of the precursor exhibited a fast sensing process with a response and recovery period of 2 s and 3 s, respectively^5^. The 3D structures of Ni-doped SnO_2_, such as hollow spheres^22^, microflowers^20^, or other hierarchical nanostructures^23^, were produced by the hydrothermal method or chemical solution route, which successfully improved the response with excellent selectivity for ethanol detection. To date, ethanol testing is needed not only for drunk driving and alcohol brewing but also for the production of biochemical products. It is imperative that researchers carry out significant work on the sensitivity, selectivity, and long-term stability of ethanol sensors. However, it should be noted that the moisture resistance is often the most-overlooked aspect of gas sensors in actual use scenarios. On the other hand, NiO-doped SnO_2_ hierarchical nanostructures could be applied to reduce the influence of environmental humidity and demonstrate a fast response time and excellent gas response^24^. Even so, it is still necessary to further clarify the state of the NiO (dopant or individual phase) added to SnO_2_ nanostructures because this may extend our understanding of their gas sensing mechanisms. It is also well known that NiO shows a high affinity for water molecule absorption^25,26^.

This work reports the synthesis of NiO-decorated SnO_2_ nanosheets by a facile two-step hydrothermal process. The effects of NiO content on the structural, morphological, and gas sensing properties of SnO_2_ nanosheet-based sensors were analyzed in detail. The gas sensing results confirmed that the NiO-decorated sensors indeed exhibited highly sensitive and selective ethanol sensing properties, with excellent long-term stability and reproducibility. In particular, the 3 mol% NiO-decorated sensor had a remarkable enhancement in moisture resistance compared with the pure SnO_2_ sensor, which makes it more promising for practical application.

## Results and discussion

### Structural and morphological characteristics

**Fig. 1:**
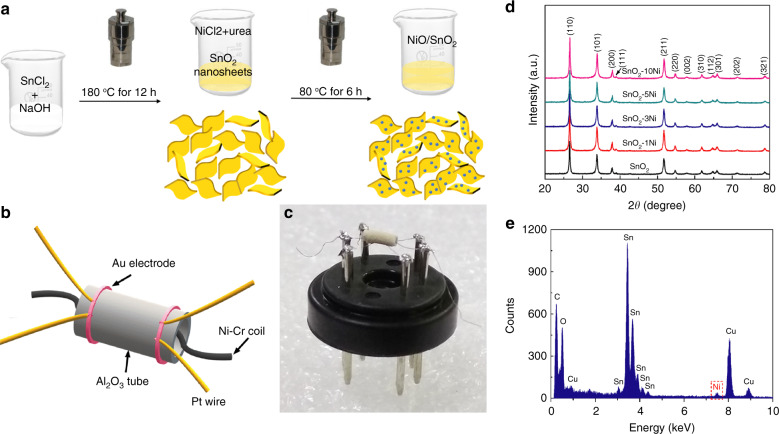
Synthesis of NiO-decorated SnO nanosheets. **a** Schematic illustration of the synthesis route for NiO-decorated SnO_2_ nanosheets; **b** Schematic diagram and **c** photograph of the as-fabricated sensor; **d** XRD patterns of the synthesized samples of pure SnO_2_ and NiO-decorated SnO_2_ nanosheets with NiO concentrations of 1, 3, 5, and 10 mol%; **e** EDX spectrum of SnO_2_-3Ni

As illustrated in Fig. 1a, SnO_2_ nanosheets can be easily decorated with NiO nanoparticles during the preparation procedure. First, precipitates were formed immediately when the SnCl_2_·2H_2_O was put into deionized water because of Sn^2+^ hydrolysis. The added NaOH also reacted with Sn^2+^ ions and accelerated its hydrolysis. Hence, the solution turned slightly white at first. The oxidation of the Sn(OH)_2_ precipitates occurred at conditions with a high pressure and high temperature of 180 °C. Following the so-called “oriented attachment” mechanism, excessive OH^−^ ions preferred to attach on the (110) of rutile SnO_2_ and bind relatively weakly to (001)^27,28^. With the control of the pH value (pH = 13), the basic units gradually aggregated to form the SnO_2_ nanosheets and grew along the [110] direction. In the secondary hydrothermal process, urea was used to ensure the homogeneous precipitation of Ni(OH)_2_ on the surface of 2D SnO_2_ nanosheets. After annealing at 500 °C in air, the NiO nanoparticle-decorated SnO_2_ nanosheets were obtained.

The crystal structures of the pure and NiO-decorated SnO_2_ samples were analyzed by X-ray diffraction (XRD), as shown in Fig. 1d. All diffraction peaks observed in the curves were indexed to tetragonal rutile SnO_2_ (JCPDS No. 41-1445). However, the NiO phase cannot be detected in these XRD patterns even though the content of NiO reaches 10 mol%. A similar phenomenon was previously reported and was explained by the low content (the second phase can be observed by XRD experiments above 40 mol%) and small crystal size of the NiO^20,29^. On the other hand, no shifts can be observed in the peaks of the NiO-decorated samples, indicating that the added Ni may not be incorporated in the SnO_2_ lattice^30^. The energy dispersive X-ray (EDX) spectrum of SnO_2_-3Ni, shown in Fig. 1e, shows that the content of Ni was approximately 2.9%, which agrees well with the intended value (3 mol%). It should be noted that the peaks for Cu and C shown in Fig. 1e originated from the copper grid in the TEM specimen.

**Fig. 2:**
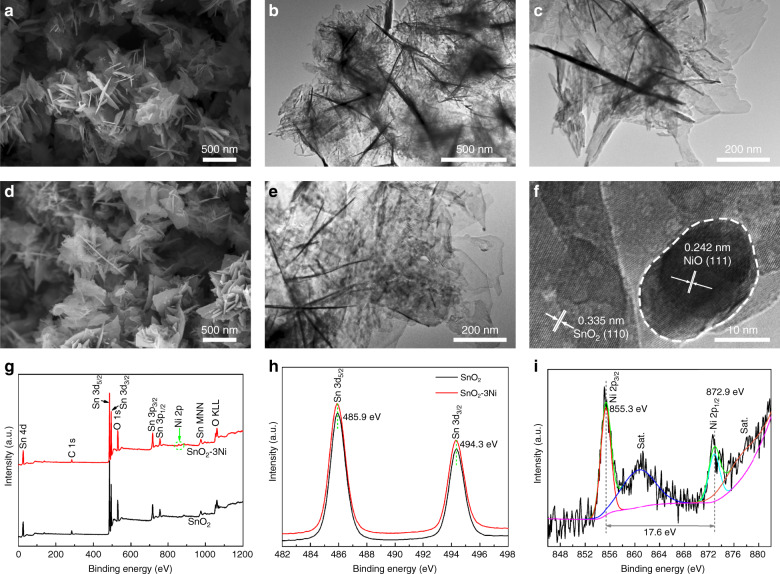
Morphologies and chemical components of pure SnO and SnO -3Ni nanosheets. **a** SEM image and **b**, **c** TEM images of pure SnO_2_ nanosheets; **d** SEM image, **e** TEM image and **f** HRTEM image of SnO_2_-3Ni nanosheets; **g** XPS survey spectra of the pure SnO_2_ and SnO_2_-3Ni samples; **h** high-resolution core level Sn 3d spectra of pure SnO_2_ nanosheets and SnO_2_-3Ni; **i** high-resolution core level Ni 2p spectra of SnO_2_-3Ni

Figure 2a–c display the scanning electron microscopy (SEM) and the transmission electron microscopy (TEM) images of the pure SnO_2_ sample. The nanosheets were in the size range of 100-500 nm with a smooth surface morphology. Compared with the pure SnO_2_ nanosheets, the 3 mol% NiO-decorated nanosheets (Fig. 2d–f) had rough surfaces and diverse shapes, which might be due to the decoration of the NiO. More details for the morphologies of the SnO_2_ samples with NiO decoration amounts of 1 mol%, 5 mol%, and 10 mol% are shown in Fig. S1. It can be clearly observed that the 2D nanosheet structure of pure SnO_2_ was well maintained for all the samples.

To further confirm the decoration of the NiO nanoparticles, we investigated the SnO_2_-3Ni nanosheets with high-resolution TEM (HRTEM), as shown in Fig. 2f. The HRTEM image demonstrates the presence of independent phases of NiO nanoparticles on the surface of the SnO_2_ nanosheets. The lattice fringes with d-spacings of 0.242 nm and 0.335 nm were obtained, which match well with the (111) plane of NiO and the (110) plane of rutile SnO_2_, respectively^5^.

X-ray photoelectron spectroscopy (XPS) was conducted to further investigate the surface compositions and the chemical states of the pure SnO_2_ and SnO_2_-3Ni nanosheets. The survey spectrum in Fig. 2g confirmed the presence of Sn, O, and C in both samples and Ni only for the SnO_2_-3Ni, where C is commonly known as an impurity component in XPS measurements. The high-resolution spectrum of Sn 3d is shown in Fig. 2h, and the peaks are consistent in the two samples. Two peaks of 485.9 eV and 494.3 eV were attributed to spin-orbit components of Sn 3d_5/2_ and Sn 3d_3/2_, respectively, corresponding to Sn^4+^ in a tetragonal rutile structure. The same binding energy of Sn 3d in the two samples suggests the formation of NiO/SnO_2_ rather than Ni-doped in SnO_2_. The core-level Ni 2p spectra are shown in Fig. 2i, where peaks at 855.3 eV and 872.9 eV were assigned to Ni 2p_3/2_ and Ni 2p_1/2_, respectively, and a spin-orbit splitting of 17.6 eV can be seen between the Ni 2p_3/2_ and Ni 2p_1/2_ peaks. The Ni 2p_3/2_ peaks were attributed to NiO_5_ or a Ni^2+^ pyramidal symmetry, according to previous literature reports^5,31^. Based on the findings above, the core level Ni 2p spectra further confirmed the formation of NiO decoration on the SnO_2_ nanosheets.

### Gas-sensing properties

**Fig. 3:**
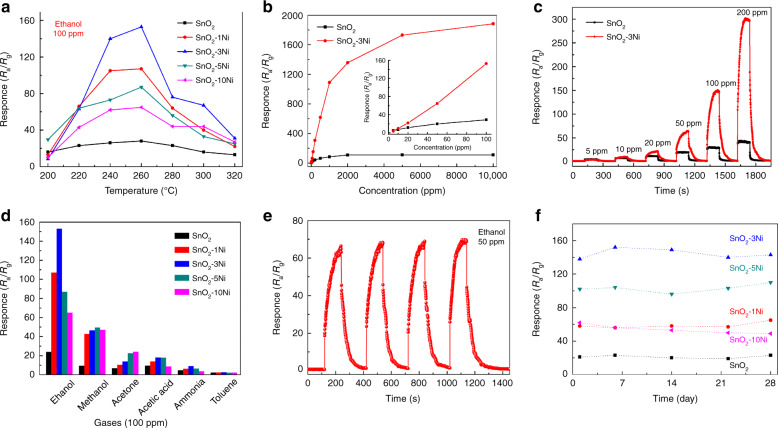
Gas-sensing properties of sensors based on pure and NiO-decorated SnO nanosheets. **a** Responses of the sensors based on pure and NiO-decorated SnO_2_ nanosheets to 100 ppm ethanol at different temperatures (200‒320 °C); **b** the responses of the sensors based on pure SnO_2_ and SnO_2_-3Ni nanosheets to various ethanol concentrations (5‒10,000 ppm) at 260 °C, where the inset shows the response at low concentrations (5–100 ppm); **c** typical response-recovery curves of the sensors to ethanol correspond to the inset of (**b**); **d** selectivity of the sensors to 100 ppm gases at 260 °C; **e** the response curve of SnO_2_-3Ni based sensor to 50 ppm ethanol at 260 °C (four cycles); **f** long-term stability of the sensors to 100 ppm ethanol at 260 °C for 4 weeks

To verify the optimum operating temperature, the responses of the sensors based on pure and NiO-decorated SnO_2_ nanosheets to 100 ppm ethanol were investigated from 200 to 320 °C, as shown in Fig. 3a. For all sensors, the response first increased, reached a maximum value at an optimum operating temperature, and decreased with increasing temperature. Obviously, the optimum operating temperature of all the sensors was approximately 260 °C. It is worth noting that all the NiO-decorated sensors exhibited significantly improved ethanol sensing properties compared with the pure SnO_2_-based sensor. In particular, the SnO_2_-3Ni sensor exhibited the best performance of the samples considered in this study, and a high response of 153 was achieved at 260 °C. We also noticed that an excessive amount of NiO decoration resulted in a decrease in the response. The responses of sensors based on SnO_2_, SnO_2_-1Ni, SnO_2_-3Ni, SnO_2_-5Ni and SnO_2_-10Ni (at 260 °C) were 28, 107, 153, 87, and 65, respectively.

Figure 3b displays the response of the pure SnO_2_ and SnO_2_-3Ni-based sensors to ethanol with concentrations ranging from 5 to 10000 ppm. The response of the pure SnO_2_ sensor had a significant increase at the ethanol concentration below 500 ppm and then tended to saturate at 2000 ppm. In comparison, the response of the SnO_2_-3Ni sensor increased rapidly in the range of 5‒2000 ppm and then continued to increase with ethanol concentration up to 10,000 ppm, which suggests a higher maximum detection range for the SnO_2_-3Ni sensor than for the pure SnO_2_. The inset curves of Fig. 3b demonstrate a magnified view of the responses at low concentrations (5–100 ppm). Both sensors can detect low concentrations of ethanol down to 5 ppm, while the SnO_2_-3Ni-based sensor demonstrated a substantially increased response above 20 ppm. The corresponding dynamic response-recovery curves of the two sensors are shown in Fig. 3c. The response increased sharply once the sensor was exposed to ethanol and returned to its original value after exposure to air.

Another critical factor to meet the practical demands required for gas sensors is the selectivity for different gases. As shown in Fig. 3d, the sensor responses to various gases were measured at 260 °C with a fixed concentration of 100 ppm. All the sensors showed the highest response to ethanol among the six gases. For instance, the responses of the SnO_2_-3Ni sensor were 153, 46.5, 18.0, 13.9, 9.0, and 2.7 to ethanol, methanol, acetone, acetic acid, ammonia, and toluene, respectively. In other words, the SnO_2_-3Ni sensor demonstrated a good selectivity to ethanol gas.

Reproducibility and long-term stability are important requirements for the practical application of gas sensors. Figure 3e displays the response curve of the SnO_2_-3Ni sensor towards 50 ppm ethanol and contains the measurement of four continuous cycles at 260 °C. The response curves were repeated well during the four cyclic measurements, reflecting its good reproducibility. In addition, the response values of all sensors were measured for four weeks. As shown in Fig. 3f, all response values of the sensors remained around their initial value with little fluctuation during the 4-week measurement period. The response of the SnO_2_-3Ni sensor was maintained at 143 after 4 weeks.

**Fig. 4:**
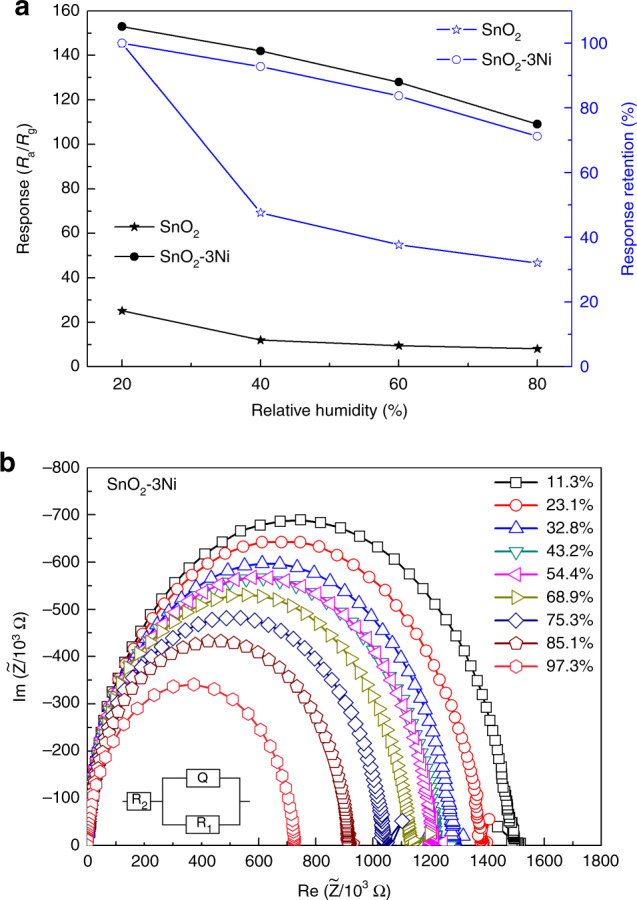
Moisture resistance of pure SnO and SnO -3Ni sensors. **a** The responses of the sensors based on pure SnO_2_ and SnO_2_-3Ni at humidity range from 20% to 80% *RH*; **b** EIS plots of the SnO_2_-3Ni sensor under different humidity conditions

The effect of humidity is a major concern for the performance and stability of SnO_2_-based gas sensors^2,14,24^. As shown in Fig. 4a, the response of the sensor based on SnO_2_-3Ni maintained 71% of its initial value when the relative humidity increased from 20% to 80% *RH*, while that of the sensor based on pure SnO_2_ decreased to 32%. This comparison indicates that the resistance of a gas sensor to a humid environment could be significantly improved with the help of NiO nanoparticles. To investigate the impact of humidity on the sensors based on SnO_2_ nanosheets decorated with NiO, the SnO_2_-3Ni sensors were analyzed by electrochemical impedance spectroscopy (EIS) under various humidities at 260 °C (the optimum operating temperature) during the measurement. As shown in Fig. 4b, the semicircles were fitted by an equivalent QR model (shown insert of Fig. 4b). The value of *R*_1_ extracted from the semicircles was influenced by humidity. Additionally, *Q* is a small phase element that was almost constant during the investigation, and *R*_2_ is the contact resistance during the measurement and far less than that of *R*_1_. With increasing *RH*, *R*_1_ decreased when the water molecule reacted with the absorbed oxygen species. The EIS plots in Fig. S2 show the same tendency, which confirms our assumption and the QR model. The major difference is that the resistance of the SnO_2_-3Ni was much larger than that of the pure SnO_2_. As mentioned in Fig. 4a, the SnO_2_-3Ni sensor maintained a high response to ethanol in an environment with high relative humidity. However, Figure S3 also shows that the moisture resistance of the SnO_2_-3Ni sensor was mainly determined by its resistance change in air (*R*_a_). The EIS plots can directly indicate a change in *R*_a_, to some extent, and reflect the moisture resistance of the gas sensors.

### Gas-sensing mechanisms

**Fig. 5:**
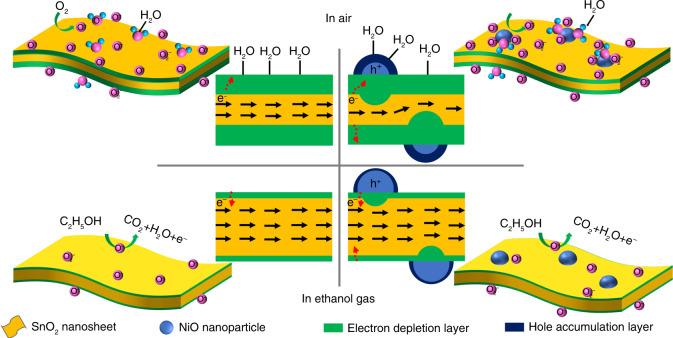
Ethanol sensing mechanisms of pure and NiO-decorated SnO nanosheets. Schematic illustration of ethanol sensing mechanisms of pure (left) and NiO-decorated (right) SnO_2_ nanosheets

The gas-sensing mechanism of SnO_2_ (an n-type MOX) has been generally explained as a resistance change resulting from the gas absorption-dissociation on the surface of the sensing material. The absorbed oxygen molecules ionize to oxygen species (O_2_^−^, O^−^, and O^2−^) by capturing the electrons from the conduction band of the SnO_2_ in air. Once exposed to ethanol vapor, the ethanol molecules react with the absorbed oxygen species, which results in thin electron depletion layers that decrease the resistance. Figure 5 compares the ethanol sensing mechanisms of the NiO-decorated SnO_2_ nanosheets with those of pure SnO_2_ nanosheets. As previously reported, p–n heterojunctions at the interface between the NiO and SnO_2_ bend the bands of p-type and n-type semiconductors in the depletion layers, resulting in the equalization of Fermi levels^5,16,32,33^. In air, both electrons in the conduction band of SnO_2_ and holes in the valance band of NiO ionize the absorbed oxygen molecules, which broadens the width of electron depletion layers on the surface of SnO_2_ nanosheets and hole accumulation layers on the surface of NiO nanoparticles. It should be noted that the NiO-decorated sensors presented a higher sensor resistance in air (*R*_a_) than that of pure SnO_2_-based sensors at the same operating temperatures. When the sensors were exposed to ethanol gas, the electrons, resulting from the reaction between ethanol molecules and oxygen species, passed through the NiO/SnO_2_ interface attributed to a p–n heterojunction. The electron depletion layer and hole accumulation layer became narrow, which led to a broader conductive channel in the SnO_2_ nanosheets and decreased the sensor resistance (*R*_g_).

We can also see that an excessive amount of NiO decoration led to a decrease in the response. The reason may be explained as follows. (1) An excessive amount of NiO further broadens the width of the electron depletion region between a NiO nanoparticle and the SnO_2_ nanosheet, making it difficult to adjust the electron transfer in the SnO_2_ nanosheets. (2) As a p-type MOX, an excessive amount of NiO also captures partial free electrons during the ethanol sensing process, which hinders the decrease in *R*_g_. Consequently, an appropriate amount of NiO is of great importance to promote the sensor performance of NiO/SnO_2_. On the other hand, the selectivity of the sensor is always affected by the operating temperature (or determined by the ratio of the absorbed oxygen species to the target gases). In this work, NiO-decorated SnO_2_ sensors show a strong catalytic capacity to ethanol at 260 °C, which requires further discussion^34^. In addition, NiO could act as a catalyst to facilitate the oxidation reaction on the surface of SnO_2_ nanosheets^23,33^. The amount of oxygen adsorbed on NiO is markedly larger than that of SnO_2_ due to charge compensation through the oxidation of Ni^2+^ to Ni^3+^^26^. Considering the more efficient carrier regulatory mechanisms with the help of NiO decoration, the NiO-decorated SnO_2_ nanosheets indeed exhibited improved ethanol sensing properties.

When the sensor operated in a high *RH* environment, there were many oxygen species absorbed onto the NiO nanoparticles, which interacted with the water molecules, providing a good response to ethanol. Moreover, NiO was more capable of adsorbing water molecules than SnO_2_^25,26^. Therefore, the SnO_2_ nanosheets decorated with NiO nanoparticles maintained an excellent ethanol sensing performance with little response loss in a high *RH* environment due to the NiO-SnO_2_ p–n heterojunctions and the increased oxidation reaction facilitated by the NiO decoration.

## Conclusions

In summary, tetragonal rutile SnO_2_ nanosheets decorated with NiO nanoparticles were successfully prepared by a template-free two-step hydrothermal method. The SnO_2_ nanosheets decorated with NiO nanoparticles exhibited excellent sensing performance towards ethanol detection. With an optimum NiO decoration amount of 3 mol%, a high response of 153 was achieved to 100 ppm ethanol gas at 260 °C, compared to 28 for the sensor with the pure SnO_2_ nanosheets. All the sensors demonstrated good selectivity of ethanol to other reductive gases (methanol, acetone, acetic acid, ammonia, and toluene), good reproducibility, and excellent long-term stability. These findings were attributed to a p–n junction forming between the NiO nanoparticles and SnO_2_ nanosheets. The SnO_2_-3Ni sensor also exhibited high moisture resistance in a high *RH* environment. Hence, SnO_2_ nanosheets decorated with NiO nanoparticles are promising candidates for ethanol sensing applications.

## Materials and methods

### Synthesis of NiO-decorated SnO_2_ nanosheets

All the reagents were of analytical grade and were used without any further purification. NiO-decorated SnO_2_ nanosheets were obtained by a two-step hydrothermal process, as illustrated in Fig. 1a. In the first step, 6 mmol SnCl_2_·2H_2_O was dissolved into 20 mL of deionized water. Then, the solution was adjusted to pH = 13 with 0.4 M NaOH solution. The mixture was stirred for 30 min and transferred into a 50 mL Teflon-lined stainless autoclave. The autoclave was sealed and kept in an oven at 180 °C for 12 h and cooled naturally to room temperature. The SnO_2_ nanosheets were collected by centrifugation and successively washed with deionized water and absolute ethanol several times to remove any residual ions and finally dried at 80 °C overnight^27^. In the second step, the as-obtained powder (0.1 g) was fully dispersed in 20 mL deionized water with sonication. A certain amount of nickel chloride (NiCl_2_, 0.2 M) solution and urea (molar ratio NiCl_2_: urea = 1:10) were added to the above suspension under continuous magnetic stirring. Then, the mixture was transferred into autoclave again and maintained at 80 °C for 6 h. The final product was collected and washed, as described previously, and calcined at 500 °C for 2 h in air. For comparison, SnO_2_ nanosheets with different contents of NiO (1, 3, 5, and 10 mol%) were prepared and referred to as SnO_2_-1Ni, SnO_2_-3Ni, SnO_2_-5Ni, and SnO_2_-10Ni, respectively.

### Characterization

X-ray diffraction (XRD) patterns were recorded on an X-ray diffractometer (Rigaku Smartlab) using Cu *K*_α_ radiation. The morphologies of the samples were characterized by scanning electron microscopy (SEM, Zeiss Gemini) and high-resolution transmission electron microscopy (HRTEM, FEI Tecnai G2 F30), where the high-resolution transmission electron microscope was equipped with energy dispersive X-ray spectroscopy (EDX). X-ray photoelectron spectroscopy (XPS) was carried out on ESCALAB 250Xi.

### Fabrication and sensor measurement

Gas-sensing measurements were performed on a commercial WS-30B system (Weisheng Instruments Co., Zhengzhou, China). Figure 1b displays a schematic diagram of the ceramic tube device used in our gas sensing measurements. Two ring-shaped Au electrodes were pasted at each end of the Al_2_O_3_ tube as the testing electrodes, and each Au electrode was connected with two Pt wires. A Ni-Cr coil was placed inside the tube to control the operating temperature. Figure 1c displays a photograph of the as-fabricated sensor with SnO_2_-based materials coated on the Al_2_O_3_ tube. In brief, the as-obtained products were mixed with a proper amount of binder (ethylcellulose: terpinol = 10:90 wt%) and pasted onto the Al_2_O_3_ tube^35^. After drying at 80 °C, all sensors were heated at 400 °C for 2 h in air. During the test, the operating temperature varied from 200 to 320 °C at a constant humidity of 20% *RH*. The gas response is defined as *R*_a_/*R*_g_ (*R*_a_: sensor resistance in air, and *R*_g_: sensor resistance in the target gas). Impedance measurements were characterized by the E4990A impedance analyzer (Agilent Tech., Inc.). The heating power was supported by a PWS2721 DC Power Supply (Tektronix, Inc.). Different *RH* conditions were given by saturated salt solutions at room temperature; specifically, 11.3%, 23.1%, 33.1%, 43.2%, 55.9%, 69.9%, 75.5%, 85.1%, and 97.6% *RH* were generated by the saturated solution of LiCl, CH_3_COOK, MgCl_2_, K_2_CO_3_, Mg(NO_3_)_2_, KI, NaCl, KCl, and K_2_SO_4_, respectively^36^.

## Supplementary information

Revised Supplemental material
