# Supplementary material for: NiO nanoparticle-decorated SnO2 nanosheets for ethanol sensing with enhanced moisture resistance
Source: Microsyst Nanoeng. 2019 May 20;5:21. doi: 10.1038/s41378-019-0060-7 (PMC6526161; doi:10.1038/s41378-019-0060-7)
Supplement: Supplementary file 1 — Revised Supplemental material [file 41378_2019_60_MOESM1_ESM.doc]

**Supplementary Information**

NiO nanoparticle-decorated SnO_2_ nanosheets for ethanol sensing with enhanced moisture resistance

Gaoqiang Niu^1,2^, Changhui Zhao^1,2^*, Huimin Gong^1^, Zhitao Yang^1^, Xiaohui Leng^1,2^, and Fei Wang^1,2^*

^1^School of Microelectronics, Southern University of Science and Technology, Shenzhen 518055, China

^2^Department of Electrical and Electronic Engineering, Southern University of Science and Technology, Shenzhen 518055, China

Corresponding Authors

*E-mails: wangf@sustc.edu.cn; zhaoch@sustc.edu.cn.

**Contents**

**Figure S1.** SEM images of (a) SnO_2_-1Ni (b) SnO_2_-5Ni (c) SnO_2_-10Ni.

**Figure S2.** EIS plots of the sensor based on pure SnO_2_ in different humidity conditions.

**Figure S3.** Sensor resistances of (a) SnO_2_ and (b) SnO_2_-3Ni in air or in 100 ppm ethanol under different *RH* conditions.

**
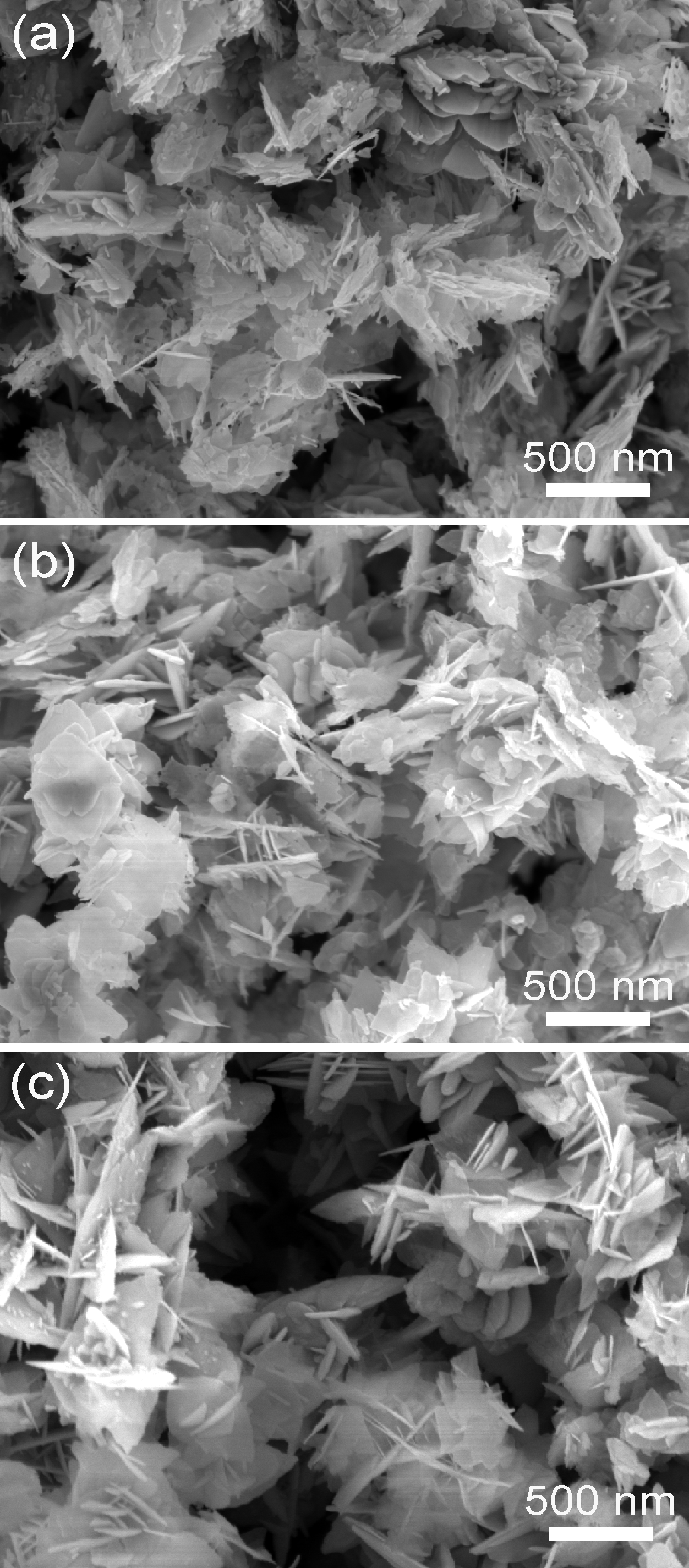
**

**Figure S1.** SEM images of (a) SnO_2_-1Ni (b) SnO_2_-5Ni (c) SnO_2_-10Ni.


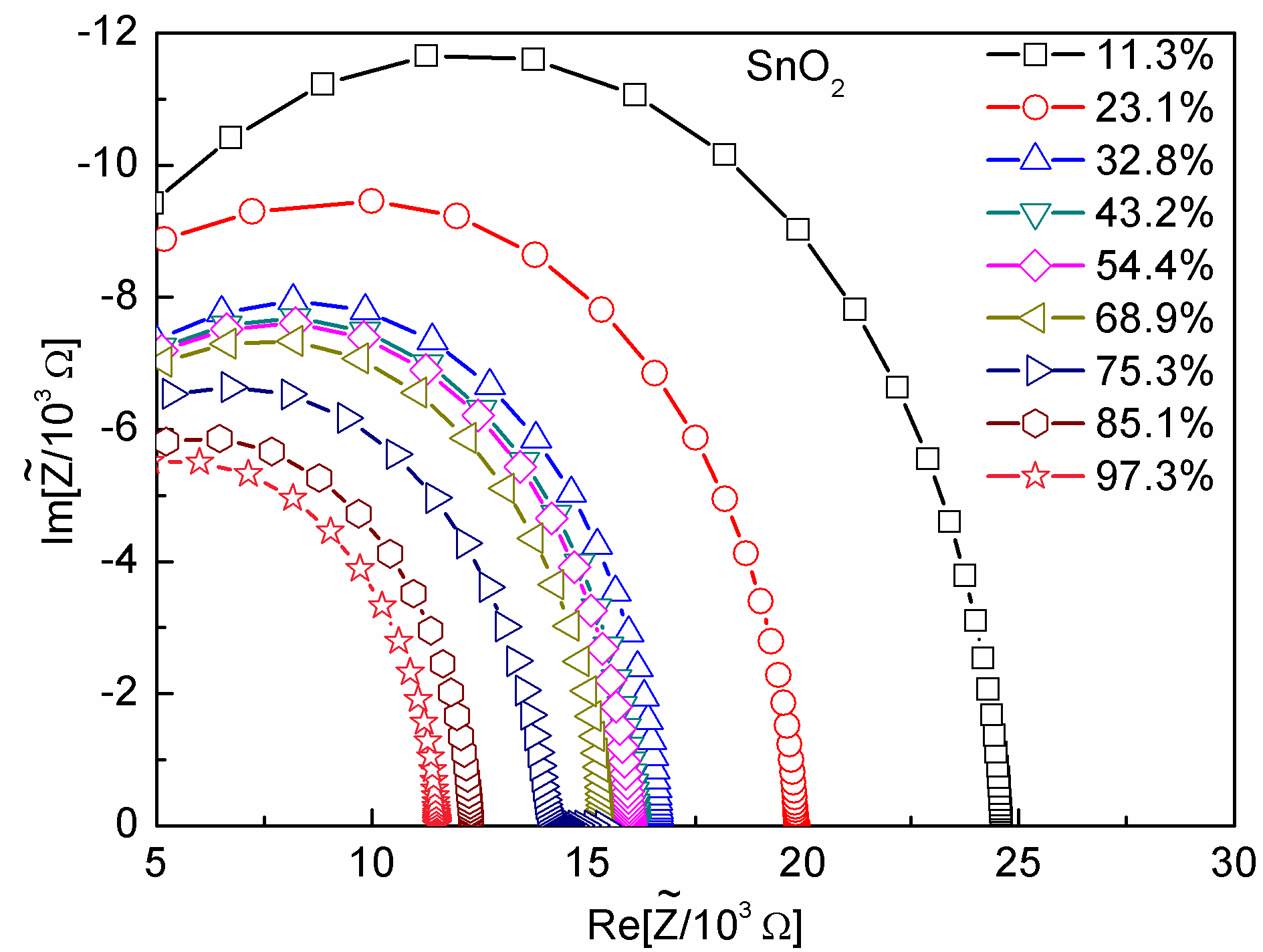


**Figure S2.** EIS plots of the sensor based on pure SnO_2_ under different humidity conditions.


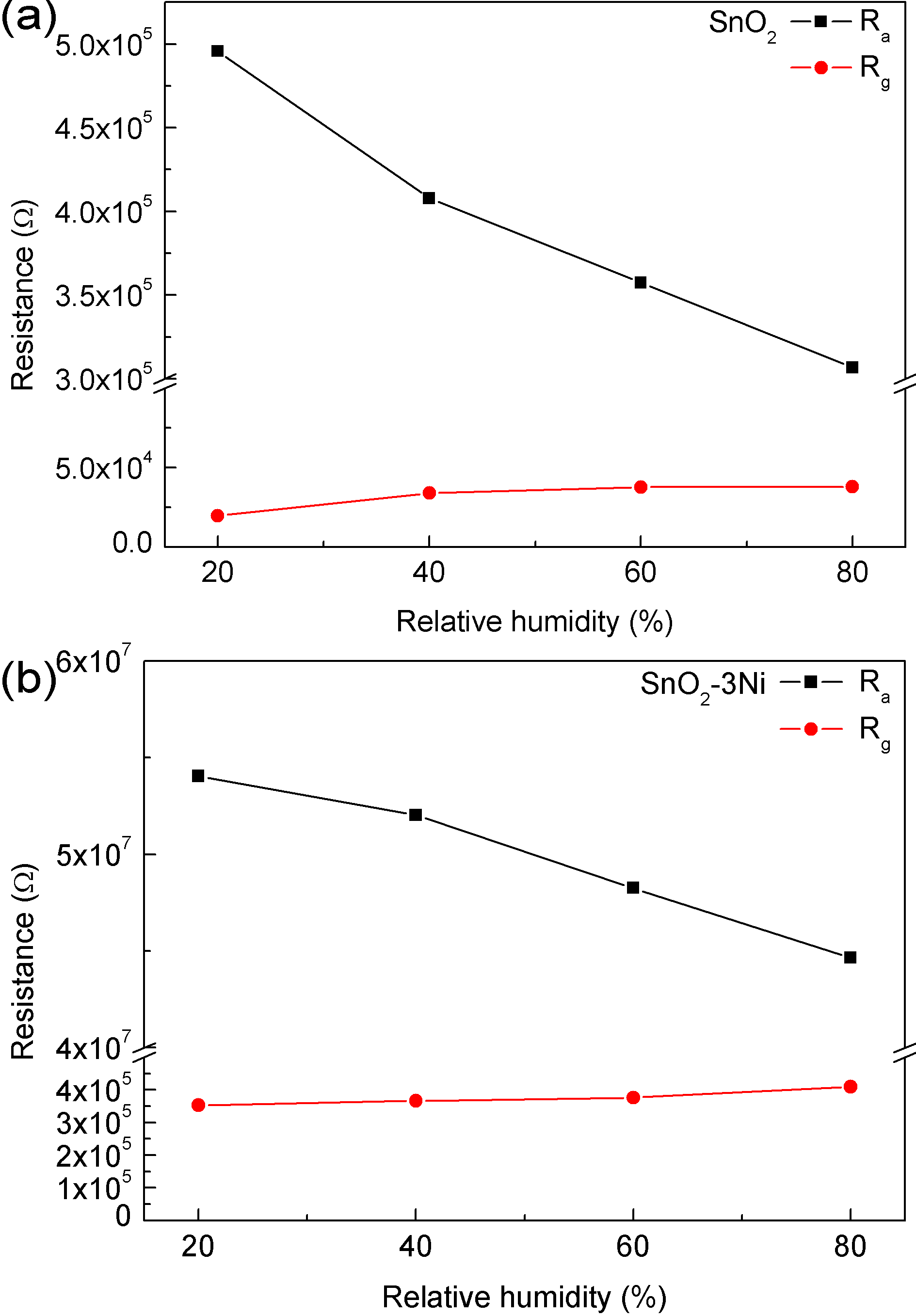


**Figure S3.** Sensor resistances of (a) SnO_2_ and (b) SnO_2_-3Ni in air or in 100 ppm ethanol under different RH conditions.
